# Length of hospital stay is associated with a decline in activities of daily living in hemodialysis patients: a prospective cohort study

**DOI:** 10.1186/s12882-019-1674-6

**Published:** 2020-01-08

**Authors:** Tsukasa Kamitani, Shingo Fukuma, Sayaka Shimizu, Tadao Akizawa, Shunichi Fukuhara

**Affiliations:** 1 Department of Healthcare Epidemiology, School of Public Health in the Graduate School of Medicine, Kyoto University, Yoshida-Konoe-cho, Sakyo-ku, Kyoto, 606-8501 Japan; 2 Human Health Sciences, Graduate School of Medicine, Kyoto University, 53 Kawahara-cho, Shogoin, Sakyo-ku, Kyoto, 606-8507 Japan; 3 Department of Medicine, Division of Nephrology, Showa University School of Medicine, 1-5-8 Hatanodai Shinagawa-ku, Tokyo, 142-8555 Japan; 4 Center for Innovative Research for Communities and Clinical Excellence (CiRCLE), Fukushima Medical University, 1 Hikarigaoka, Fukushima, 960-1295 Japan

**Keywords:** Hemodialysis, Length of hospital stay, Activity of daily living

## Abstract

**Background:**

The impact of length of hospital stay on activities of daily living (ADLs) has not specifically been investigated among dialysis patients. Therefore, we attempt to verify the association between the length of hospital stay and the decline in ADLs among hemodialysis patients.

**Methods:**

This prospective cohort study used data from the Japanese Dialysis Outcomes and Practice Patterns Study (J-DOPPS). We included 2442 hemodialysis patients aged ≥40 years from the J-DOPPS phase V (2012–2015) and subsequently excluded those who had already lost basic activities of daily living (BADLs) as demonstrated by dependency in at least three of the five BADLs at baseline and for whom changes in ADLs had been evaluated for less than 90 days. The main exposure was the cumulative length of hospital stay during the follow-up period. The primary outcomes were a decline in at least one of the five BADLs and eight instrumental activities of daily living (IADLs). We compared risk ratios (RRs) for 30-day increments for hospital stays with 10-year increments for age and having diabetes.

**Results:**

A total of 849 patients were included in the statistical analysis. The cumulative length of hospital stay was significantly associated with a risk of decline in ADLs (adjusted RRs [95% confidence intervals] per 30-day increments: 1.42 [1.15 to 1.75] for BADLs, 1.38 [1.13 to 1.68] for IADLs). The adjusted RRs [95% CI] for 10-year increments in age were 1.20 [0.96 to 1.50] and 1.21 [1.00 to 1.47]. The adjusted RRs [95% CI] for having diabetes were 1.36 [0.97 to 1.91] for BADLs and 1.38 [1.04 to 1.84] for IADLs.

**Conclusion:**

The impact of a 30-day increment in the cumulative length of hospital stay on the decline in ADLs was comparable to that of a 10-year increase in age and having diabetes.

## Background

Functional impairment is a strong predictor of quality of life and mortality among dialysis patients, as well as the general population [1, 2]. Among patients with end-stage renal disease, the prevalence of functional impairment is exceedingly high [3, 4]. Dependency in instrumental activities of daily living (IADLs) and basic activities of daily living (BADLs) poses problems for dialysis patients, in particular—for instance, impairments in taking medication or independently managing transportation can reduce self-care and prevent patients from visiting the clinic for dialysis [4]. An earlier study reported that more than 50% of older dialysis patients exhibited dependency in IADLs and BADLs, and only 5% were completely independent in doing both types of activities [3]. However, a remarkable decline in physical function is frequently observed even in younger (i.e., those under 65 years of age) hemodialysis patients. Another study reported that approximately 60% of patients over 21 years, who had been receiving maintenance dialysis, needed assistance at least 1 of the 4 BADLs and the 7 IADLs [5]. Therefore, dependency in IADLs and BADLs (i.e., functional impairment) should be paid attention even in relatively younger hemodialysis patients.

Hospitalization is an important risk factor influencing the degree of functional decline, particularly in older adults [6–9]. Several earlier studies have demonstrated that more than 30% of older patients developed new or additional dependencies in BADLs during a hospital stay [10, 11]. Another study demonstrated that the number of hospitalizations had a dose-response relationship with functional decline [8]. From a practical perspective, the strategies used to reduce the number of hospitalizations might not clear-cut for hemodialysis patients, since they frequently have to visit hospitals to ensure effective care management (e.g., management of vascular access). For this population, reducing the total length of hospital stay might be a more realistic target. To our knowledge, no study has evaluated the association between length of hospital stay and functional impairment in hemodialysis patients, in particular, to date. Thus, in the current study, we aim to clarify the associations of the length of hospital stay and the number of hospitalizations with declines in BADLs and IADLs.

## Methods

### Study population, design, and setting

This prospective cohort study used data from the Japanese Dialysis Outcomes and Practice Patterns Study (J-DOPPS). It is a part of the Dialysis Outcomes and Practice Patterns Study (DOPPS), which is an international longitudinal study conducted on hemodialysis patients. The patients included in the J-DOPPS were randomly selected from some representative dialysis facilities in Japan. Their demographic information, laboratory data, comorbidities, dialysis conditions, medication (assessed every 4 months), and information on hospitalization and death were collected. All patients provided written, informed consent at the time of study enrollment. More details on the DOPPS are available in the literature [12].

In this study, we included 2442 patients aged ≥40 years, who participated in J-DOPPS phase V (2012–2015). As we tried to assess the decline in BADLs as our primary outcome, we excluded all patients who had already lost BADLs as was operationalized by dependency in at least three of the five BADLs at baseline. This criterion was used, as such patients would not be likely to be considered at-risk for a further decline in BADLs. In addition, patients for whom changes in ADLs were evaluated for less than 90 days, in which the change in ADLs will not occur sufficiently, were also excluded.

### Measurements

#### Hospitalization

We identified the occurrence of hospitalization based on the participants’ medical records. Only those hospitalizations with more than a two-day hospital stay were identified because one-day hospitalizations were assumed not to influence the outcomes. We recorded the length of hospital stay for each hospitalization and the number of hospitalizations for each patient. The cumulative length of hospital stay and number of hospitalizations were used as exposure variables in the statistical analysis.

#### Change in activities of daily livings

We evaluated BADLs and IADLs using self-report questionnaires. BADLs were assessed using the Katz index [13]. Using this tool, participants answered whether they could independently perform five tasks (i.e., they answered either independent or not for each task). IADLs were assessed using the Lawton-Brody IADL scale [14], which asks participants to evaluate their ability to perform eight tasks on a 3-point scale (need no help, need some help, or unable to do at all). BADLs and IADLs were assessed twice: while registering for the J-DOPPS Phase V (baseline) and during follow-up the next year. The outcomes were defined as a decline in any one of the five BADLs and eight IADLs from baseline to follow-up.

#### Other variables

We collected information on the participants’ age, sex, body mass index (BMI), smoking behavior, and dialysis vintage (years on hemodialysis, from initiation to the baseline survey). BMI was categorized into < 18.5 kg/m^2^, ≥18.5 to < 25 kg/m^2^, and ≥ 25 kg/m^2^. We also obtained information on the presence of comorbidities (e.g., diabetes, cerebrovascular diseases, coronary heart disease, other cardiovascular diseases, congestive heart disease, cancer other than skin cancer, neurological disease, peripheral vascular disease, dementia, and psychiatric disorder) from participants’ medical records. Further, the most recent laboratory data on albumin, phosphorus, creatinine, single-pool Kt/V, pre-dialysis blood urea nitrogen, and hemoglobin levels were obtained at baseline. Subsequently, we calculated a functional status score at baseline by combining the scores of the Katz index and Lawton-Brody IADL scale using an algorithm developed in a previous study [2, 15].

### Statistical analysis

We conducted the following statistical analyses only for patients without any missing data (i.e., complete case analysis). All statistical analyses were conducted using Stata 15.1 (StataCorp, College Station, TX).

In the descriptive analysis, we described the participants’ baseline characteristics according to their hospitalization status; we used means and standard deviations (SDs) or medians and interquartile ranges (IQRs) for continuous variables and the number and proportion for categorical variables. The distribution of cumulative length of hospital stay among the patients who had been hospitalized was depicted in a histogram. The proportion of decline in each BADL and IADL according to hospitalization status was depicted in a bar graph.

To clarify the association between hospitalization and declines in ADLs, we calculated the risk ratio (RR) and 95% confidence interval (CI) from the mean predicted probabilities based on a fitted logit model, in which the estimated coefficients are transformed into probabilities through a logistic function. The RR can then be calculated as the ratio of the estimated probabilities, using the user-written command “adjrr” in Stata [16].

In the primary analysis, the cumulative length of hospital stay was set as the independent variable. Then, we separately calculated the RRs for one-day increments of hospital stays and 95% CIs for the decline in BADLs and IADLs with adjustments for potential confounding factors (i.e., age, sex, dialysis vintage, BMI, functional status score, comorbidities, albumin, phosphorus, creatinine and single-pool Kt/V). We used cluster-robust variance to consider cluster effects according to the facility. For a simple interpretation of the impact of the exposure on the outcomes, we compared the RRs of 7- and 30-day increments for hospital stays with 10-year increments for age and having diabetes. A restricted cubic spline curve analysis with three knots was used to visually confirm the linear or nonlinear relationship between the cumulative length of hospital stay and predicted probabilities of decline in BADLs and IADLs. For this analysis, we employed logistic regression models, with adjustment for the same confounding factors as in primary analysis.

For the secondary analysis, we first categorized the number of hospitalizations into three categories (0, 1, or ≥ 2) and used it as an independent variable (with 0 as the reference) in the same statistical model as was used in the primary analysis. Second, we conducted the aforementioned two analyses by age group (< 65, ≥ 65 years) to verify whether the magnitude of the association between hospitalization and decline in ADLs differs between older and younger patients. To test the interaction, we added the product term for the cumulative length of hospitalization and age group for the primary analytic model and that of the number of hospitalizations and age group for the secondary analytic model. The statistical significance of the product terms was evaluated using a Wald test. Third, we conducted analyses by the two dialysis vintage groups that were classified based on ≥5 years (the closest value to the median) or < 5 years, as well as the age group. Fourth, we conducted an analysis using infection-, cardiovascular disease- and vascular access-related hospitalization as the exposure variables. We evaluated the associations between these cause-specific hospitalizations and a decline in ADLs.

Finally, we conducted three sensitivity analyses to confirm the robustness of the primary analysis. First, we defined the outcomes as a decline in two of the five BADLs and eight IADLs. Second, since the different evaluation periods for the change in ADLs for each patient (ranging from 93 to 566 days) could affect the results, we analyzed only those patients with an evaluation period within the IQR (338 to 376 days). Finally, we included 148 patients who were previously excluded due to dependency as measured by having at least three of the five BADLs during the primary analysis. For all analyses, *p* < 0.05 was considered statistically significant.

## Results

### Study population

**Fig. 1:**
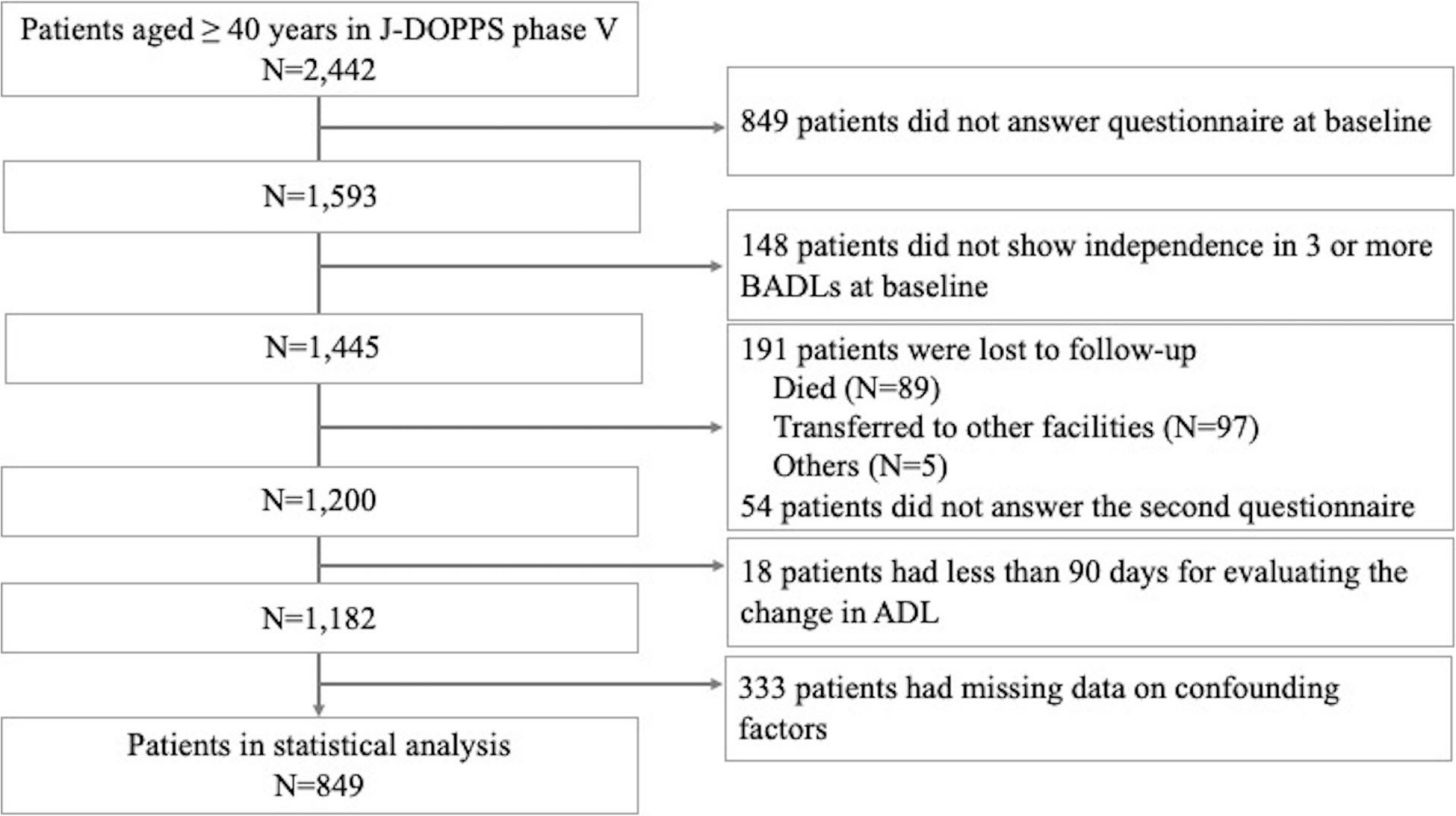
The flowchart of study participants

Figure 1 shows the flow of the study participants. A total of 2442 patients aged ≥40 years participated in J-DOPPS Phase V. Among the 1593 patients who answered the questionnaire at baseline, 148 were excluded because they were not independent in more than three of the five BADLs at baseline. In the follow-up survey, 1200 patients answered the questionnaire. After excluding 18 patients with an evaluation period for the change in ADLs of less 90 days, 849 patients who had no missing data were included in the statistical analysis.

**Fig. 2:**
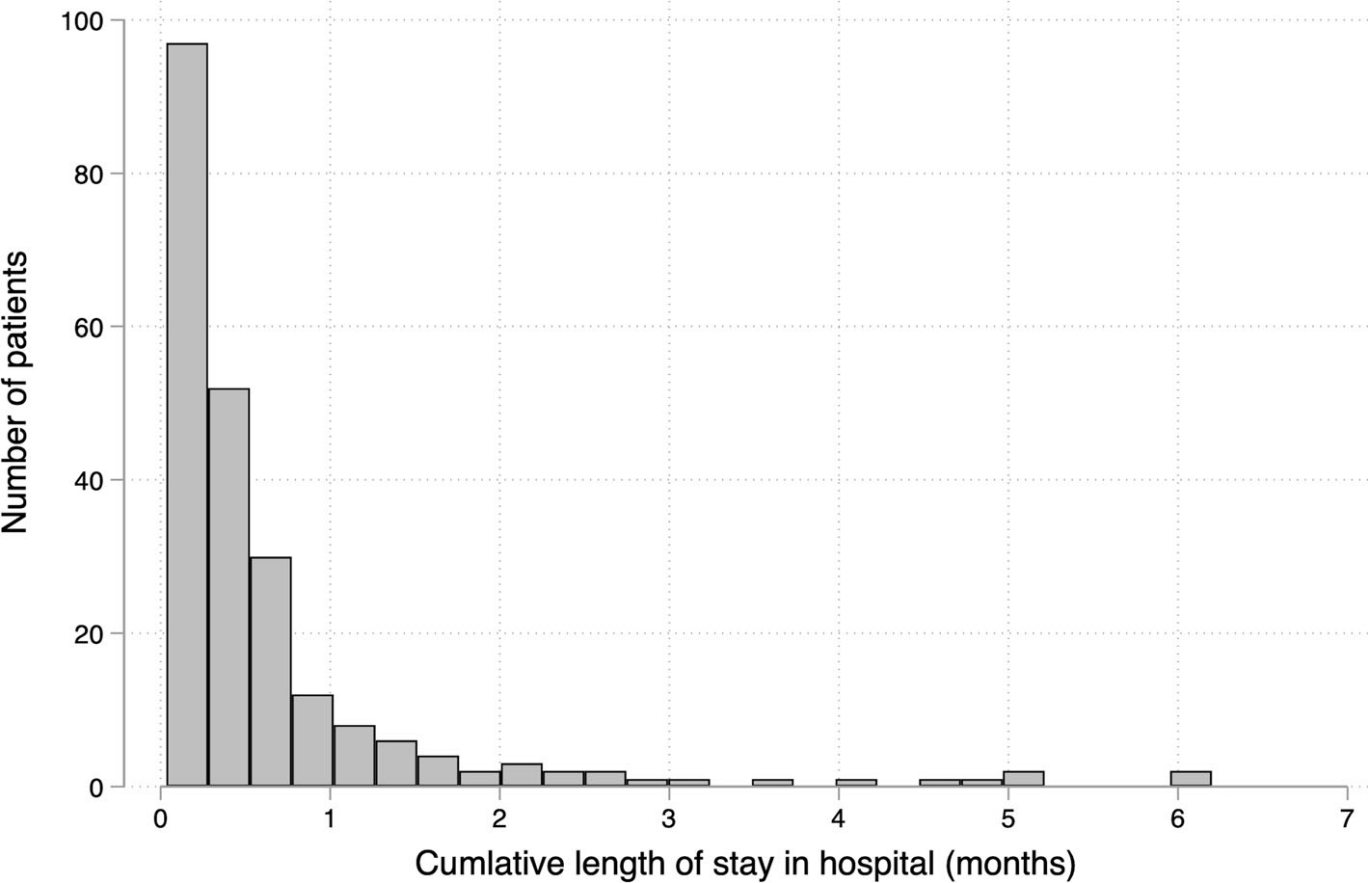
Distribution of the cumulative length of hospital stay

Table 1 depicts the baseline characteristics of the 849 participants included in the statistical analysis. Their mean age was 64.9 years (SD, 10.7), and 64.4% of the participants were men. The proportion of patients with diabetes was 36.3%. During the follow-up period (median, 359 days; IQR, 337–375 days), 228 (26.9%) patients experienced at least one hospitalization with a stay of more than 2 days. Figure 2 depicts the distribution of the cumulative length of hospital stay among patients who had been hospitalized. The median of the cumulative length of hospital stay was 11 days (range 2–187). A decline in BADLs and IADLs occurred in 143 (16.8%) and 198 (23.3%) patients from the baseline and follow-up, respectively.

**Table 1:** Baseline characteristics of study participants

	Total (*n* = 849)	Non-hospitalized Participants (*n* = 621)	Hospitalized Participants (*n* = 228)
Age (years), mean [SD]	64.9 [10.7]	64.3 [10.8]	66.7 [10.1]
Male, n (%)	547 (64.4)	397 (63.9)	150 (65.8)
Dialysis vintage (years), median [IQR]	5.6 [2.2–11.6]	5.5 [2.1–11.4]	5.6 [2.3–12.6]
BMI (kg/m^2^), n (%)			
< 18.5	176 (20.7)	120 (19.3)	56 (24.6)
≥ 18.5 to < 25	559 (65.8)	416 (67.0)	143 (62.7)
≥ 25	114 (13.4)	85 (13.7)	29 (12.7)
Functional status score, n (%)			
< 8	49 (5.8)	34 (5.5)	15 (6.6)
8 to < 11	77 (9.1)	55 (8.9)	22 (9.6)
11 to < 13	171 (20.1)	108 (17.4)	63 (27.6)
13	552 (65.0)	424 (68.3)	128 (56.1)
Comorbidities, n (%)			
Diabetes	308 (36.3)	212 (34.1)	96 (42.1)
Cerebrovascular diseases	71 (8.4)	49 (7.9)	22 (9.6)
Coronary heart disease	202 (23.8)	138 (22.2)	64 (28.1)
Other cardiovascular diseases	175 (20.6)	118 (19.0)	57 (25.0)
Congestive heart disease	131 (15.4)	89 (14.3)	42 (18.4)
Cancer other than skin cancer	84 (9.9)	61 (9.8)	23 (10.1)
Neurological disease	35 (4.1)	25 (4.0)	10 (4.4)
Peripheral vascular disease	110 (13.0)	65 (10.5)	45 (19.7)
Dementia	3 (0.4)	2 (0.3)	1 (0.4)
Psychiatric disorder	32 (3.8)	21 (3.4)	11 (4.8)
Albumin (g/dL), mean [SD]	3.7 [0.4]	3.7 [0.4]	3.6 [0.4]
Phosphorus (mg/dL), mean [SD]	5.3 [1.3]	5.3 [1.3]	5.3 [1.4]
Creatinine (mg/dL), mean [SD]	10.7 [2.5]	10.9 [2.5]	10.4 [2.5]
Blood urea nitrogen (mg/dL), mean [SD]	63.8 [14.1]	64.1 [14.1]	63.1 [14.1]
Hemoglobin (g/L), mean [SD]	10.6 [1.1]	10.6 [1.1]	10.5 [1.3]
Single-pool Kt/V, mean [SD]	1.4 [0.3]	1.4 [0.3]	1.4 [0.3]

SD, standard deviation; IQR, interquartile range;

### Proportion of decline for each BADL and IADL

**Fig. 3:**
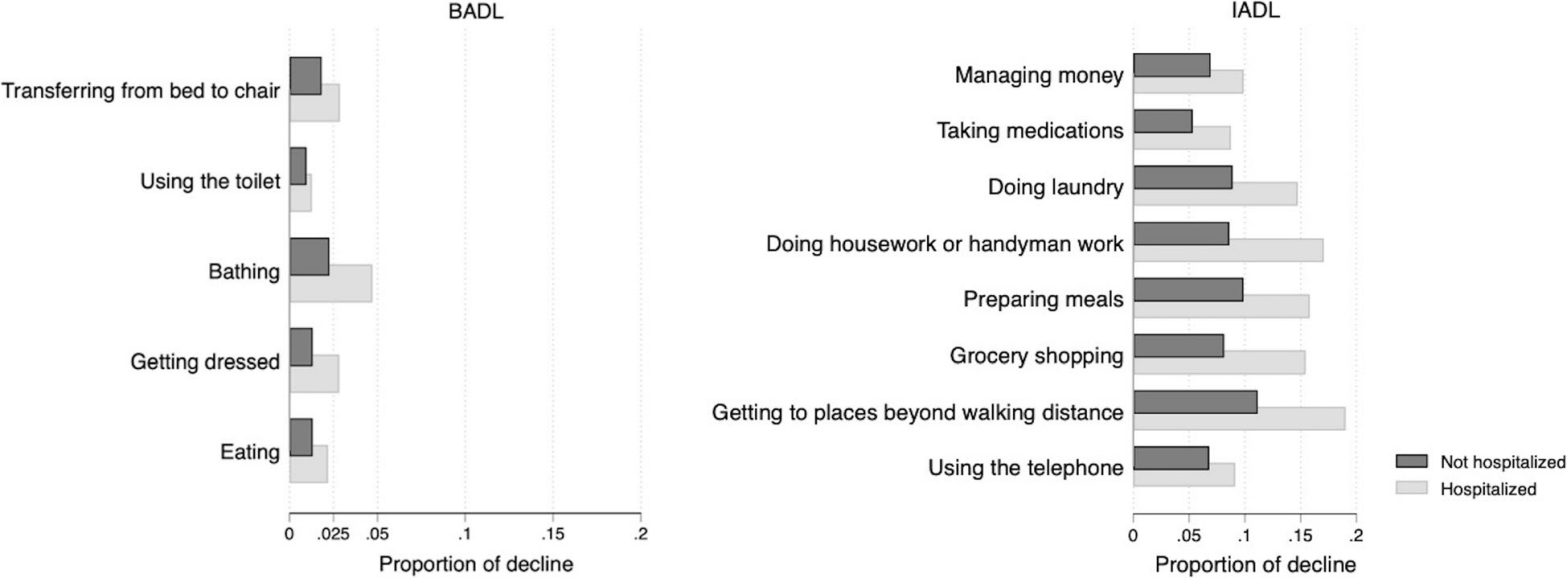
Proportions of declines in BADLs and IADLs according to hospitalization BADL, basic activities of daily living; IADL, instrumental activities of daily living

Figure 3 depicts the proportion of BADLs and IADLs, showing a decline according to patients’ hospitalization status. Among the BADLs, a decline in bathing was most frequently observed (4%), whereas a decline in using the toilet was least frequently observed (1%) in the hospitalized patients. Among the IADLs, 20% of the hospitalized patients showed a decline in getting to places beyond walking distance. Even the IADLs showing the lowest proportions of decline (i.e., taking medications and using the telephone) had higher proportions than bathing (a BADL). For all BADLs and IADLs, the proportion of decline was higher among hospitalized than non-hospitalized patients.

### Association between cumulative length of hospital stay and declines in BADLs and IADLs

**Fig. 4:**
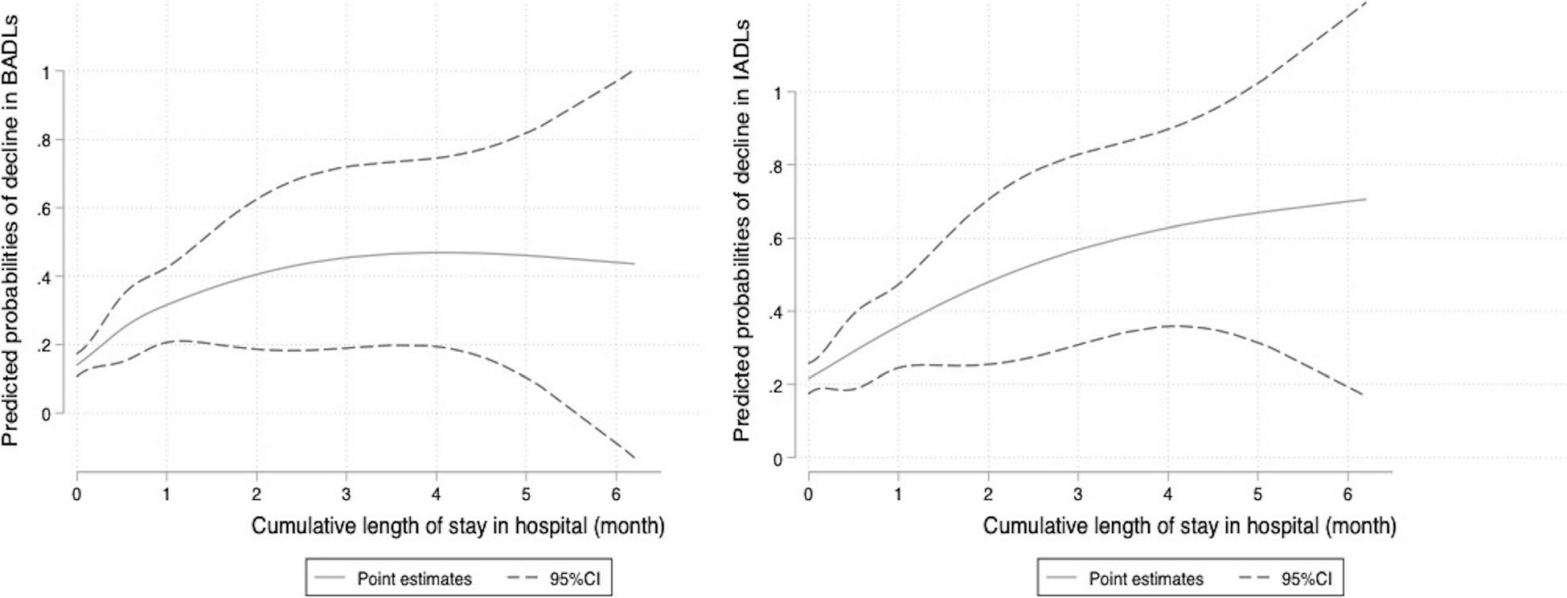
Linear relationship between cumulative length of hospital stay and decline in ADLs. BADL, basic activities of daily living; IADL, instrumental activities of daily living; CI, confidence interval. Adjusted for age, sex, dialysis vintage, body mass index (< 18.5, ≥18.5 to < 25, ≥25), functional status score, comorbidities (diabetes, cerebrovascular diseases, coronary heart disease, other cardiovascular diseases, congestive heart disease, cancer other than skin cancer, neurologic disease, peripheral vascular disease, dementia, and psychiatric disorders), albumin, phosphorus, creatinine and single-pool Kt/V

The cumulative length of hospital stay was associated with an increased risk of decline in BADLs (adjusted RR (per day), 1.012, 95% CI [1.005 to 1.020], *P* = 0.002) and IADLs (adjusted RR (per day), 1.011, 95% CI [1.004 to 1.019], *P* = 0.003). For age (per 10-year increment), the RRs [95% CI] of declines in BADLs and IADLs were 1.20, 95% CI [0.96–1.50] (*P* = 0.176) and 1.21, 95% CI [1.00 to 1.47] (*P* = 0.046), respectively. Regarding diabetes, the RRs [95% CI] for declines were 1.36, 95% CI [0.97 to 1.91] (*P* = 0.072) for BADLs and 1.38, 95% CI [1.04 to 1.84] (*P* = 0.027) for IADLs (Table 2). A restricted cubic spline curve analysis showed the linear relationships between the cumulative length of hospital stay and predicted probabilities of decline in BADLs and IADLs using the restricted cubic spline curve (Fig. 4).

**Table 2:** Association between cumulative length of hospital stay and decline in ADLs

	BADLs		IADLs	
	Crude RR [95% CI]	Adjusted RR^a^ [95% CI]	Crude RR [95% CI]	Adjusted RR^a^ [95% CI]
Cumulative length of hospital stay				
Per day	1.014 [1.006 to 1.022]	1.012 [1.005 to 1.020]	1.015 [1.007 to 1.023]	1.011 [1.004 to 1.019]
Per 7 days	1.10 [1.04 to 1.16]	1.12 [1.04 to 1.19]	1.11 [1.05 to 1.17]	1.08 [1.03 to 1.14]
Per 30 days	1.47 [1.18 to 1.83]	1.42 [1.15 to 1.75]	1.51 [1.23 to 1.85]	1.38 [1.13 to 1.68]
Age (per 10 years)		1.20 [0.96 to 1.50]		1.21 [1.00 to 1.47]
Diabetes		1.36 [0.97 to 1.91]		1.38 [1.04 to 1.84]

ADL, activities of daily living; BADL, basic activities of daily living; IADL, instrumental activities of daily living; RR, risk ratio; CI, confidence interval

^a^Length of hospital stay, age and diabetes were included simultaneously in the model after adjusting for sex, dialysis vintage, body mass index (< 18.5, ≥ 18.5 to < 25, ≥ 25), functional status score, comorbidities (cerebrovascular diseases, coronary heart disease, other cardiovascular diseases, congestive heart disease, cancer other than skin cancer, neurologic disease, peripheral vascular disease, dementia, and psychiatric disorder), albumin, phosphorus, creatinine and single-pool Kt/V

### Association between number of hospitalizations and decline in BADLs and IADLs

The incidences of decline in BADLs according to the number of hospitalizations categorized as 0, 1, and ≥ 2 were 13.0% (*n* = 81/621), 22.9% (*n* = 32/140), and 34.1% (*n* = 30/88), respectively, whereas the incidences of decline in IADLs were 20.0% (*n* = 124/621), 30.7% (*n* = 43/140), and 35.2% (*n* = 31/88), respectively. The adjusted RRs for declines in BADLs among patients with one hospitalization and for those with more than two hospitalizations were 1.69, 95% CI [1.17 to 2.45] (*P* = 0.006) and 2.27, 95% CI [1.58 to 3.26] (*P* < 0.001), respectively, compared to patients without hospitalization. The adjusted RRs [95% CI] for declines in IADLs in patients with one and more than two hospitalizations were 1.41, 95% CI [1.09 to 1.83] (*P* = 0.008) and 1.39, 95% CI [0.97 to 1.99] (*P* = 0.074), respectively (Table 3).

**Table 3:** Association between number of hospitalizations and decline in ADLs

	BADLs			IADLs		
	Incidence	Crude RR [95% CI]	Adjusted RR^a^ [95% CI]	Incidence	Crude RR [95% CI]	Adjusted RR^a^ [95% CI]
Number of hospitalizations						
0	13.0% (81/621)	ref	ref	20.0% (124/621)	ref	ref
1	22.9% (32/140)	1.75 [1.18 to 2.61]	1.69 [1.17 to 2.45]	30.7% (43/140)	1.54 [1.15 to 2.06]	1.41 [1.09 to 1.83]
≥ 2	34.1% (30/88)	2.61 [1.82 to 3.75]	2.27 [1.58 to 3.26]	35.2% (31/88)	1.76 [1.29 to 2.41]	1.39 [0.97 to 1.99]

BADL, basic activities of daily living; IADL, instrumental activities of daily living; RR, risk ratio; CI, confidence interval; ref., reference

^a^Adjusted for age, sex, dialysis vintage, body mass index (< 18.5, ≥18.5 to < 25, ≥25), functional status score, comorbidities (diabetes, cerebrovascular diseases, coronary heart disease, other cardiovascular diseases, congestive heart disease, cancer other than skin cancer, neurologic disease, peripheral vascular disease, dementia, and psychiatric disorder), albumin, phosphorus, creatinine and single-pool Kt/V

### Association between hospitalization and decline in BADLs and IADLs by age group

The incidences of decline in BADLs and IADLs were 13.7 and 18.7% among patients aged under 65 years and 19.6 and 27.5% among patients aged 65 years or older, respectively. The adjusted RRs for declines in BADLs and IADLs per 1 day increment in the cumulative length of hospital stay were 1.013, 95% CI [1.001 to 1.025] (*P* = 0.034) and 1.017, 95% CI [1.006 to 1.028] (*P* = 0.003), respectively, among patients aged below 65 years, whereas they were 1.014, 95% CI [1.003 to 1.026] (*P* = 0.017) and 1.007, 95% CI [0.999 to 1.015] (*P* = 0.112), respectively, among patients aged 65 years or older (p for interaction = 0.52 for BADLs and 0.43 for IADLs). Among patients aged below 65 years, the adjusted RRs [95% CI] for declines in BADLs and IADLs were 1.83, 95% CI [1.00 to 3.35] (*P* = 0.512) and 1.53, 95% CI [0.96 to 2.42] (*P* = 0.072) among patients with one hospitalization and 2.65, 95% CI [1.49 to 4.70] (*P* < 0.001) and 1.38, 95% CI [0.82 to 2.32] (*P* = 0.23) among those with more than two hospitalizations, respectively, compared to patients without a hospitalization. Among patients aged 65 years or older, the adjusted RRs for declines in BADLs and IADLs were 1.56, 95% CI [1.00 to 2.43] (*P* = 0.051) and 1.30, 95% CI [0.96 to 1.77] (*P* = 0.087) among patients with one hospitalization and 2.04, 95% CI [1.35 to 3.07] (P < 0.001) and 1.32, 95% CI [0.85 to 2.06] (*P* = 0.22) among those with more than two hospitalizations, respectively. The interactions were not statistically significant (P for interaction = 0.82 for BADLs and 0.87 for IADLs; Table 4). The results from the other secondary analyses by dialysis vintage group and cause-specific hospitalizations are shown in Additional file 1: Table S1, Additional file 1: Table S2.

**Table 4:** Association between hospitalization and decline in ADLs by age group

	BADLs		IADLs	
	< 65 years (*n* = 401)	≥65 years (n = 448)	< 65 years (*n* = 401)	≥65 years (*n* = 448)
	Adjusted RR^a^ [95% CI]			
Cumulative length of hospital stay (per day)	1.013 [1.001 to 1.025]	1.014 [1.003 to 1.026]	1.017 [1.006 to 1.028]	1.007 [0.999 to 1.015]
	P for interaction^b^ = 0.52		P for interaction^b^ = 0.43	
	BADLs		IADLs	
	< 65 years (n = 401)	≥65 years (n = 448)	< 65 years (n = 401)	≥65 years (n = 448)
Number of hospitalizations				
0	ref	ref	ref	ref
1	1.83 [1.00 to 3.35]	1.56 [1.00 to 2.43]	1.53 [0.96 to 2.42]	1.30 [0.96 to 1.77]
≥ 2	2.65 [1.49 to 4.70]	2.04 [1.35 to 3.07]	1.38 [0.82 to 2.32]	1.32 [0.85 to 2.06]
	P for interaction^c^ = 0.82		P for interaction^c^ = 0.87	

BADL, basic activities of daily living; IADL, instrumental activities of daily living; RR, risk ratio; CI, confidence interval; ref., reference

^a^Adjusted for age, sex, dialysis vintage, body mass index (< 18.5, ≥18.5 to < 25, ≥25), functional status score, comorbidities (diabetes, cerebrovascular diseases, coronary heart disease, other cardiovascular diseases, congestive heart disease, cancer other than skin cancer, neurologic disease, peripheral vascular disease, dementia, and psychiatric disorder), albumin, phosphorus, creatinine and single-pool Kt/V

^b^Testing the statistical significance of the product terms in the cumulative length of hospitalization and age group using a Wald test

^c^Testing the statistical significance of product terms in the number of hospitalizations and age group using a Wald test

### Sensitivity analysis

On defining the outcomes as a decline in two of the five BADLs and eight IADLs, the adjusted RRs for declines in BADLs and IADLs per 1-day increment in cumulative length of hospital stay were 1.010, 95% CI [1.000 to 1.020] (*P* = 0.051) and 1.007, 95% CI [0.999 to 1.015] (*P* = 0.077), respectively. Further, on analyzing only those patients whose evaluation periods for ADL changes were within the IQR (337–375 days), the adjusted RRs [95% CI] for declines in BADLs and IADLs per 1 day increment in cumulative length of hospital stay were 1.027, 95% CI [1.012 to 1.042] (*P* < 0.001) and 1.007, 95% CI [0.999 to 1.015] (*P* = 0.076), respectively. Finally, in the analysis including 148 patients who were excluded due to dependence in at least three of the five BADLs in primary analysis, the adjusted RRs for declines in BADLs and IADLs per 1 day increment in cumulative length of hospital stay were 1.012, 95% CI [1.004 to 1.019] (*P* = 0.002) and 1.012, 95% CI [1.004 to 1.019] (*P* = 0.003), respectively.

## Discussion

This large prospective cohort study revealed that the incidence of hospitalization is significantly associated with declines in BADLs and IADLs among hemodialysis patients. Both the cumulative length of hospital stays and the number of hospitalizations had positive linear relationships with the risk of decline in ADLs. A sensitivity analysis confirmed the robustness of these results. Notably, the impact of a 30-day increase in the cumulative length of hospital stay was comparable to that of a 10-year increase in age and having diabetes. Overall, our results suggest that clinical interventions and social support aimed at reducing the length of hospital stays might be an effective method of preventing declines in ADLs among hemodialysis patients.

The most commonly observed decline in BADLs for patients who had been hospitalized was in bathing (4%). Two previous studies have found similar results, that is, bathing is the most, or second-most, common BADL impairment among dialysis patients [3, 5]. This suggests that an evaluation of participants’ ability to perform bathing could be used to detect their tendency to show a decline in BADL earlier. Regarding IADLs, the most common decline was in getting to places beyond walking distance (19%) among patients who had been hospitalized. Impairments in this ability naturally lead to difficulties in visiting dialysis facilities. This, in turn, can lead to subsequent disabilities in other IADLs or even BADLs. Therefore, in clinical practice, careful assessment of challenges with visiting the dialysis facilities should be considered.

In the age group analysis, although a similar association between hospitalization and declines in ADLs was observed among patients in both age groups, the impact of hospitalization on the IADLs in older adults was smaller than was reported for younger adults. Older adults may already have more declines in IADLs than younger adults at baseline. In our data, the mean of IADL scores at baseline were 6.7 (SD = 2.3) in younger and 5.4 (SD = 2.9) in older adults, with lower scores indicating greater dependency in IADL.

The observed declines in ADLs caused by hospitalization can be mainly attributed to the disease itself, inactivity, and environmental or lifestyle changes [17]. Inactivity, in particular, causes a rapid loss of muscle strength, physical performance, and aerobic capacity in older adults [18, 19]. These functional declines can directly lead to dependency, particularly in BADLs. Hospitalization can aggravate depressive symptoms and impair cognitive functioning [20, 21], and the concomitant declines in attention and executive function can lead to dependency in more complex IADLs. Therefore, an interdisciplinary approach might be required to prevent declines in physical and cognitive function among inpatients. Such an approach would partially involve shortening the length of hospital stays, which would help patients avoid exposure to certain risk factors and ultimately prevent dependency in ADLs. Indeed, a previous intervention study conducted on patients who had suffered a hip fracture demonstrated that accelerated discharge and home-based rehabilitation improved the recovery in ADLs [22]. However, the possible strategies to address the length of hospital stay should be different by the cause of hospitalization. Providing sufficient home-based care is commonly essential for any cause of hospitalization. In addition, the etiology, severity, and symptoms of the diseases causing hospitalization can directly influence ADLs. Thus, the preventive care and care during a hospital stay for a disease-causing hospitalization also should be considered to shorten the length of hospital stays.

Our study has several strengths. First, this is the first study to verify the association between the length of hospital stay and decline in ADLs by comparing hemodialysis patients who had and who had not been hospitalized. Although several previous studies have compared community-dwelling older adults with and without hospitalization [7, 8, 23–25], no study has specifically compared hemodialysis patients, including younger adults, in the aforementioned manner to date. This comparison is important since hospitalization appears to have a major impact on ADLs, even among younger adult patients. Second, in Japan, the variance in the length of hospital stay is much larger than that of other countries due to multiple factors, such as clinical practice pattern, insurance system, and economic status. Therefore, by using data on the length of hospital stay in Japan, we could accurately assess the impact of a prolonged length of hospital stay on the decline in ADLs. Third, patients enrolled in the J-DOPPS represented hemodialysis patients in Japan, which guarantees the generalizability of our results. In other countries, where the length of hospital stay is shorter, prolonged hospital stays among older hemodialysis patients may become a social problem associated with the aging of patients in the future. Fourth, since the information on hospitalization was based on medical records, rather than self-reports, we could obtain accurate details of patients’ length of stay in each hospitalization, which enabled an accurate assessment of the association between hospitalization and declines in ADLs.

However, our study has several limitations, as well. First, the assessment of ADLs was conducted only twice—at the time of registration and during a follow-up survey in the following year; hence, we could not assess the detailed trajectory of changes in ADLs. Second, we assessed the ADLs using self-report questionnaires. A previous study demonstrated inconsistent results among self-administered, interviewer-administered, and performance-based measures of physical performance [26]. There is a tendency for subjective assessments to overestimate difficulties in IADL relative to objective assessment [27]. Although a gold standard measure for ADL assessment does not presently exist, it is vital to take into consideration that our results were based on self-reported ADLs. It also should be noted that the Katz Index and the Lawton-Brody IADL scale have been not validated in hemodialysis patients. Third, the duration from hospitalization to the follow-up survey of ADLs varied among patients, which implies that the long- and short-term effects of hospitalization on ADLs occurred mixed in our results. Potentially, our results may overestimate and underestimate the impact of hospitalization because the proportion of patients who show ADL recovery naturally increases with the duration of discharge [10, 11]. Finally, we did not account for all the confounding factors. For example, we did not adjust for socioeconomic status and use of social support, which can affect hospitalization and ADLs.

## Conclusions

Our results suggested that the length of hospital stay had a significant impact on declines in both BADLs and IADLs among hemodialysis patients. Therefore, in these patients, attempts to shorten the length of hospital stay as much as possible might help prevent ADL declines.

## Supplementary information

**Additional file 1: Table S1.** Association between cause-specific hospitalization and decline in ADLs. **Table S2.** Association between hospitalization and decline in ADLs by dialysis vintage group

## Data Availability

The data supporting the conclusions of this article are available from the Arbor Research Collaborative for Health. However, there are restrictions to the availability of these data, which were used under license for the current study and, hence, are not publicly available. Nevertheless, data are available from the authors upon reasonable request and with the permission of Arbor Research Collaborative for Health.

## Abbreviations

ADL: Activity of Daily Living;
BADL: Basic Activity of Daily Living
BMI: Body Mass Index
CI: Confidence Interval
IADL: Instrumental Activity of Daily Living
IQR: Interquartile Range
J-DOPPS: Japanese Dialysis Outcomes and Practice Patterns Study
RR: Risk Ratio
SD: Standard Deviation
