# Supplementary material for: Length of hospital stay is associated with a decline in activities of daily living in hemodialysis patients: a prospective cohort study
Source: BMC Nephrol. 2020 Jan 8;21:9. doi: 10.1186/s12882-019-1674-6 (PMC6950813; doi:10.1186/s12882-019-1674-6)
Supplement: Supplementary file 1 — Additional file 1: Table S1. Association between cause-specific hospitalization and decline in ADLs. Table S2. Association between hospitalization and decline in ADLs by dialysis vintage group [file 12882_2019_1674_MOESM1_ESM.docx]

**Appendix**

**Table S1.** Association between cause-specific hospitalization and decline in ADLs

|  | BADLs | | IADLs | |
| --- | --- | --- | --- | --- |
|  | Adjusted RR^a^ | 95% CI | Adjusted RR^a^ | 95% CI |
| Infection |  | | | |
| No Hospitalization | ref | | ref | |
| No infection-related hospitalization | 1.82 | 1.36 to 2.44 | 1.35 | 1.06 to 1.71 |
| Infection-related hospitalization | 3.09 | 1.46 to 6.56 | 2.16 | 1.12 to 4.16 |
| Cardiovascular disease |  | | | |
| No Hospitalization | ref | | ref | |
| No CVD-related hospitalization | 1.58 | 1.15 to 2.16 | 1.29 | 1.02 to 1.62 |
| CVD-related hospitalization | 2.79 | 1.95 to 3.99 | 1.69 | 1.19 to 2.39 |
| Vascular access |  | | | |
| No Hospitalization | ref | | ref | |
| No vascular access-related hospitalization | 1.89 | 1.37 to 2.61 | 1.42 | 1.10 to 1.83 |
| Vascular access-related hospitalization | 1.87 | 1.15 to 3.02 | 1.28 | 0.86 to 1.92 |

BADL, basic activities of daily living; IADL, instrumental activities of daily living; RR, risk ratio; CI, confidence interval; ref, reference; CVD, cardiovascular disease

^a^Adjusted for age, sex, dialysis vintage, body mass index (< 18.5, ≥ 18.5 to < 25, ≥ 25), functional status score, comorbidities (diabetes, cerebrovascular diseases, coronary heart disease, other cardiovascular diseases, congestive heart disease, cancer other than skin cancer, neurologic disease, peripheral vascular disease, dementia, and psychiatric disorder), albumin, phosphorus, creatinine and single-pool Kt/V.

**Table S2.** Association between hospitalization and decline in ADLs by dialysis vintage group

|  | BADLs | | IADLs | |
| --- | --- | --- | --- | --- |
|  | Vintage < 5 years  n = 387 | Vintage ≥ 5 years  n = 462 | Vintage < 5 years  n = 387 | Vintage ≥ 5 years  n = 462 |
|  | Adjusted RR^a^ [95% CI] | | | |
| Cumulative length of hospital stay (per day) | 1.011  [1.001 to 1.022] | 1.013  [0.999 to 1.028] | 1.012  [1.003 to 1.021] | 1.011  [0.999 to 1.021] |
|  | P for interaction^b^ = 0.81 | | P for interaction^b^ = 0.98 | |
|  | | | | |
|  | BADLs | | IADLs | |
|  | Vintage < 5 years  n = 387 | Vintage ≥ 5 years  n = 462 | Vintage < 5 years  n = 387 | Vintage ≥ 5 years  n = 462 |
| Number of hospitalizations |  |  |  |  |
| 0 | ref | ref | ref | ref |
| 1 | 2.05  [1.31 to 3.22] | 1.58  [0.91 to 2.75] | 1.16  [0.79 to 1.72] | 1.65  [1.15 to 2.36] |
| ≥2 | 2.71  [1.60 to 4.59] | 1.92  [1.04 to 3.55] | 1.41  [0.91 to 2.19] | 1.36  [0.76 to 2.45] |
|  | P for interaction^c^ =0.60 | | P for interaction^c^ =0.41 | |

BADL, basic activities of daily living; IADL, instrumental activities of daily living; RR, risk ratio; CI, confidence interval; ref, reference.

^a^Adjusted for age, sex, body mass index (<18.5, ≥18.5 to <25, ≥25), functional status score, comorbidities (diabetes, cerebrovascular diseases, coronary heart disease, other cardiovascular diseases, congestive heart disease, cancer other than skin cancer, neurologic disease, peripheral vascular disease, dementia, and psychiatric disorder), albumin, phosphorus, creatinine and single-pool Kt/V.

^b^Test the statistical significance of product terms of cumulative length of hospitalization and dialysis vintage group by a Wald test.

^c^Test the statistical significance of product terms of number of hospitalizations and dialysis vintage group by a Wald test.
